# Deep semi-supervised learning for automatic segmentation of inferior alveolar nerve using a convolutional neural network

**DOI:** 10.1186/s12903-021-01983-5

**Published:** 2021-12-07

**Authors:** Ho-Kyung Lim, Seok-Ki Jung, Seung-Hyun Kim, Yongwon Cho, In-Seok Song

**Affiliations:** 1 Department of Oral and Maxillofacial Surgery, Korea University Guro Hospital, 148, Gurodong-ro, Guro-gu, Seoul, 08308 Republic of Korea; 2 Department of Orthodontics, Korea University Guro Hospital, 148, Gurodong-ro, Guro-gu, Seoul, 08308 Republic of Korea; 3 Department of Medical Humanities, Korea University College of Medicine, 46, Gaeunsa 2-gil, Seongbuk-gu, Seoul, 02842 Republic of Korea; 4 Department of Radiology and AI Center, Korea University College of Medicine, Korea University Anam Hospital, 73, Goryeodae-ro, Seongbuk-gu, Seoul, 02841 Republic of Korea; 5 Department of Oral and Maxillofacial Surgery, Korea University Anam Hospital, 73, Goryeodae-ro, Seongbuk-gu, Seoul, 02841 Republic of Korea

**Keywords:** Deep learning, Convolutional neural network, Automatic segmentation, Inferior alveolar nerve

## Abstract

**Background:**

The inferior alveolar nerve (IAN) innervates and regulates the sensation of the mandibular teeth and lower lip. The position of the IAN should be monitored prior to surgery. Therefore, a study using artificial intelligence (AI) was planned to image and track the position of the IAN automatically for a quicker and safer surgery.

**Methods:**

A total of 138 cone-beam computed tomography datasets (Internal: 98, External: 40) collected from multiple centers (three hospitals) were used in the study. A customized 3D nnU-Net was used for image segmentation. Active learning, which consists of three steps, was carried out in iterations for 83 datasets with cumulative additions after each step. Subsequently, the accuracy of the model for IAN segmentation was evaluated using the 50 datasets. The accuracy by deriving the dice similarity coefficient (DSC) value and the segmentation time for each learning step were compared. In addition, visual scoring was considered to comparatively evaluate the manual and automatic segmentation.

**Results:**

After learning, the DSC gradually increased to 0.48 ± 0.11 to 0.50 ± 0.11, and 0.58 ± 0.08. The DSC for the external dataset was 0.49 ± 0.12. The times required for segmentation were 124.8, 143.4, and 86.4 s, showing a large decrease at the final stage. In visual scoring, the accuracy of manual segmentation was found to be higher than that of automatic segmentation.

**Conclusions:**

The deep active learning framework can serve as a fast, accurate, and robust clinical tool for demarcating IAN location.

## Background

The inferior alveolar nerve (IAN) is a branch of the mandibular division of the trigeminal nerve, and it is an important anatomical structure in dentistry since it transmits the sensation in mandibular teeth and lower lip [1]. The location of the IAN influences the prognosis of an orthognathic surgery, pathological treatment of benign, or malignant, and outpatient procedures, such as dental implants and extraction [1–3]. Compared to plain radiography, cone-beam computed tomography (CBCT) can easily determine the position of the IAN. However, it is still difficult to intuitively judge the three-dimensional (3D) position, and an operator is required to determine the location. Additionally, even if the location is judged, under static IAN tracking, the possibility of nerve damage still exists [4].

Since artificial intelligence (AI) was first introduced, the deep learning technology has been advanced consistently [5, 6]. Deep learning technology using a convolutional neural network (CNN) exhibits excellent performance in image analysis. This involves object detection, which refers to finding a specific object in an image; object classification, which refers to classifying the corresponding object; and object segmentation, which refers to finding and separating the area of a specific object [7]. Presently, AI can analyze and evaluate images, which can contribute significantly in medical diagnosis [8, 9].

Therefore, the segmentation technique has been studied to determine the precise position of the IAN using deep learning technology and 3D CBCT data. If the position of the IAN is automatically detected on a CBCT image, the IAN can be dynamically tracked during the surgery, which reduces the burden on the surgeon and improves the accuracy of the procedure while making it quicker and safer.

Earlier, the statistical shape model (SSM) described by Abdolali et al. was used for automatic segmentation of the IAN [10]. However, the SSM approach requires annotations for the segmented mandible during training. Thus, additional manual annotation or algorithm development is required. In another method described by Moris et al., segmentation was performed by selecting a predefined threshold for the grayscale value of the image that best separates the IAN from other tissues [11]. However, since the Hounsfield unit (HU) scale is not accurate in CBCT scans and the results depend on the quality of the imaging device, methodological limitations exist [12].

In the present study, a stepped 3D nnU-Net method that enables active learning was utilized to increase training efficiency with limited data and reduce labeling efforts by including AI [13]. Here, a person evaluates and corrects the result obtained from an AI, and the AI learns iteratively for organic and dynamic performance improvement. In order to explore the possibility of dynamic tracking of IAN, our study aimed at determining the precise position of the IAN using AI and evaluated its accuracy by comparing the predicted position of the nerve to the position manually designated by multiple specialists. In addition, it was verified that the segmentation accuracy and annotation efficiency can be improved with active learning.

## Material and methods

### Dataset collection

A study using dataset of multi-center was conceived and conducted for the study. Data pertaining to 138 patients who visited Korea University Anam Hospital (A), Korea University Ansan Hospital (B), and Korea University Guro Hospital (C) between January 2018 and May 2020 were utilized in this study [Internal: 98 patients (A), External: 40 patients (20 each from B and C)]. Only CBCT data of adults whose mandible and inferior alveolar nerves have matured were extracted. In order to avoid the effects of marrow dystrophy, data of those aged 60 years or older were not collected. The equipment used were KAVO 3D Exam 17–19 (Imaging Sciences International, Hatfield, PA, USA) at A, CS 9300 (Carestream Dental, GA, USA) at B, and Vatech Pax-Reve3D (Vatech, Seong-Nam, Gyeonggi-do, Korea) at C (Table 1). This study was conducted in accordance with the Declaration of Helsinki under the approval of the institutional ethics committee of each hospital [approved by the Institutional Review Board of A (2020AN0410, 11/01/2021), B (2021AS0041, 09/02/2021), and C (2021GR0148, 07/04/2021)].

**Table 1:** Characteristics and data collection parameters for the study population

Characteristic	Training and tuning (A) (N = 83)	Internal validation (A) (N = 15)	External validation (B) (N = 20)	External validation (C) (N = 20)
Age (in years)	59.9 ± 17.2	63.1 ± 16.9	40.0 ± 19.7	43 ± 18.6
Male	44	8	10	10
Female	39	7	10	10
Tube voltage (kV)	120	120	90	90
Tube current (mA)	5	5	4	4
Scan time (s)	16.8	16.8	14.3	15
Voxel size (mm)	0.3	0.3	0.3	0.08 ~ 0.25
FOV (mm^2^)	230 × 170	230 × 170	170 × 135	150 × 150
Focal spot (mm)	0.58	0.58	0.70	0.50

Internal dataset: Korea University Anam Hospital (A); External dataset: Korea University Ansan Hospital (B) and Korea University Guro Hospital (C)

FOV, field-of-view

### Training architecture

**Fig. 1:**
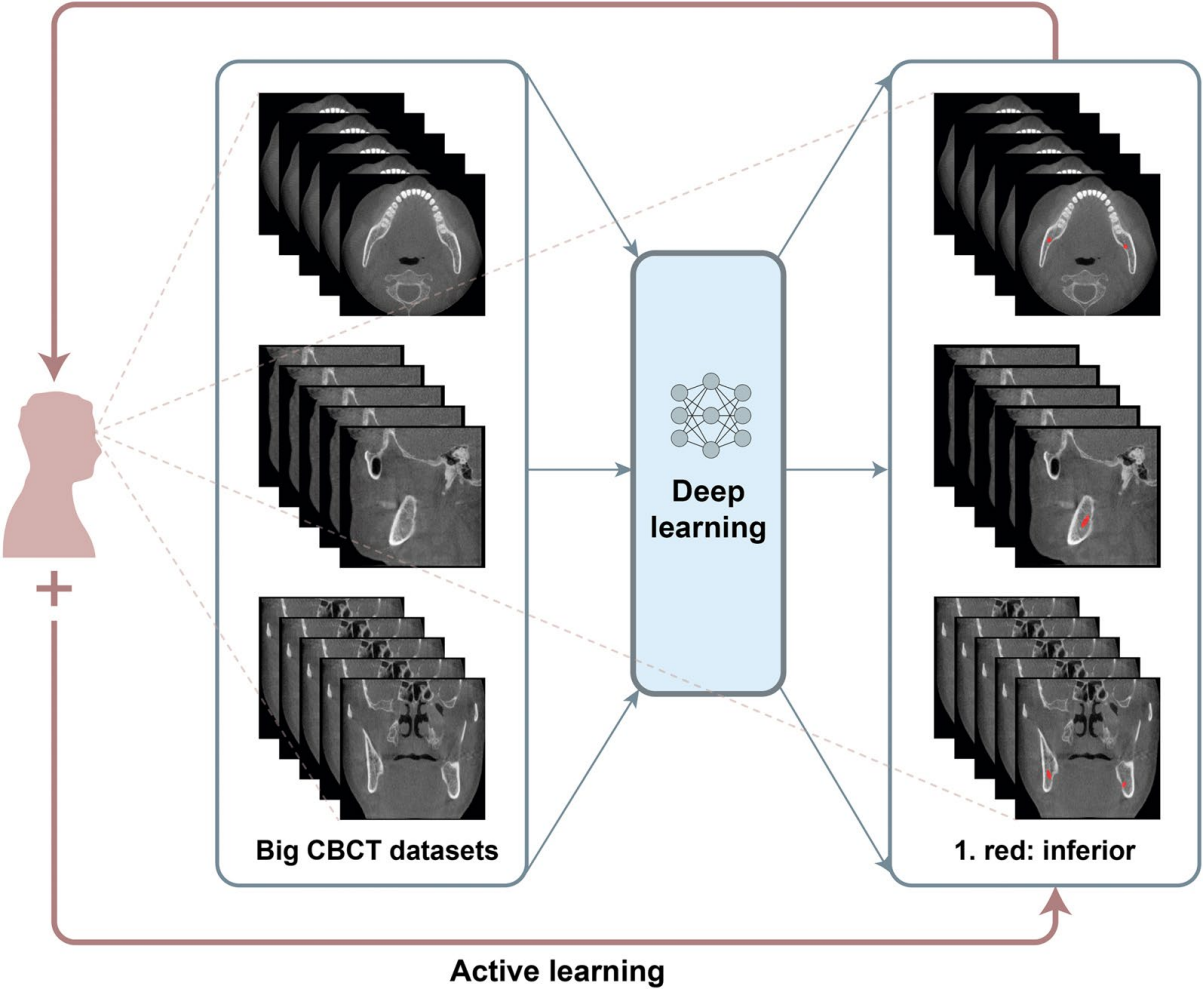
Deep learning architecture of the customized 3D U-Net adapted from nnU-Net

The overall design of the study was similar to a previous one where lesion and air of the maxillary sinus were segmented [14]. In the present study, a customized nnU-Net was used for image segmentation [13] (Fig. 1). The nnU-Net structure consists of an encoder that uses a convolutional filter and max pooling, as well as a decoder that decodes the signal back to the original dimension. The first layer of the encoder consists of 30 convolutional filters, wherein the spatial information is compressed and reduced. Further, the spatial information is restored using deconvolution by the decoder, and the information on the boundary is reinforced.

### Active learning

The active learning process comprised three steps. Here, a new dataset was added at each step while using the model learned in the previous step. This improved the model's performance as much as possible with a limited training dataset.

The internal dataset of 98 patients was randomly divided into training, tuning, and testing sets at a ratio of 7:1:2 (68 patients: 15 patients: 15 patients). Two-dimensional (2D) segmentation maps along the vertical axis of the CBCT images were constructed. The ground truth of the IAN was provided by three specialists. The overall segmentation was performed using AVIEW Modeler software (version 1.0.3, Coreline Software, Seoul, Korea). All input volumes were resized to 320 × 320 pixels, and the intensity was normalized. Data augmentation was performed using gamma adjustment, random scaling, random rotation, mirroring, and random elastic deformations. The dilation limit of the IAN thickness was set to 3 mm.

**Fig. 2:**
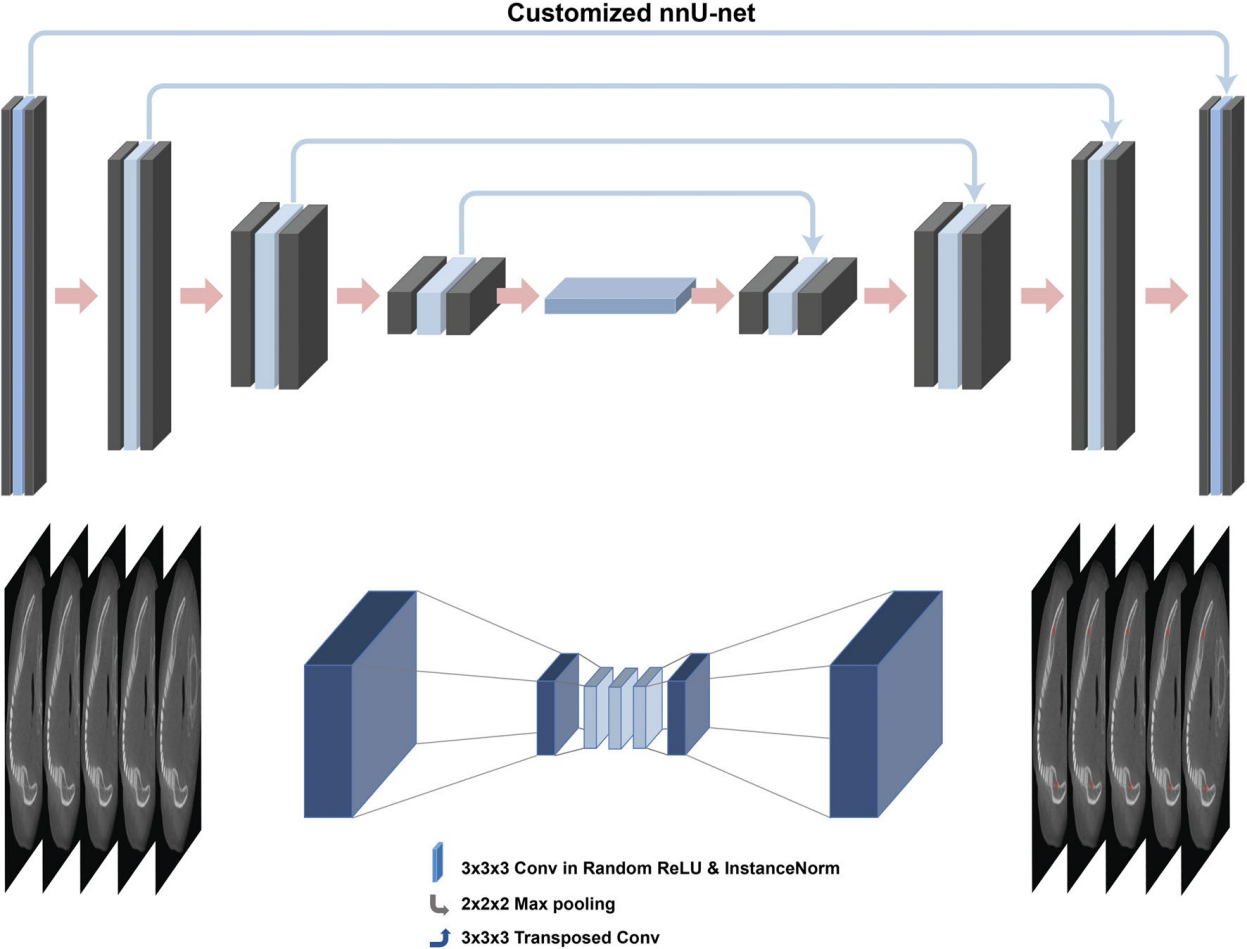
Overall active learning process for inferior alveolar nerve segmentation on cone beam computed tomography images

In the first stage, the ground truth was established by three specialists for 19 CBCT datasets from A. The AVIEW Modeler software was contained with the function of labeling the IAN in 3D view. First, the IAN was segmented automatically in the program, and then the IAN position was corrected manually on the sagittal, coronal, and axial cross-sectional images. After the labeling process, training was conducted to segment the IAN using the labeled dataset. After the first step, a new training process was conducted with 49 datasets that added 30 new unlabeled datasets. Using the model learned in the process, segmentation was first performed, and then three specialists adjusted it to determine the ground truth. In the third step, 34 new unlabeled datasets were added again, and additional training was performed using a total of 83 datasets. This process was repeated to train the model and improve its performance. Moreover, the model was tested using the remaining dataset. The accuracy of each model in IAN segmentation considering a total of 55 datasets, including the remaining 15 internal scans (A) and 40 external scans (20 each from B and C) were evaluated (Fig. 2).

### Training setup

The random rectified linear unit (ReLU) function as the activation function was used. The cross-entropy, dice coefficient, and boundary loss functions were used for training. For IAN segmentation learning, adaptive layer-instance normalization (AdaLin) and Adam optimizer were used along with an initial learning rate of 3 × 10^–4^, and a l2-weight decay value of 3 × 10^–5^. The change in the learning rate was reduced by a factor of 0.2 if the loss value did not improve during 30 epochs. Learning was terminated after 1000 epochs or when the learning rate was below 10^–6^. For optimal model selection, tuning was performed throughout the training process per an epoch using the tuning set among training set in Table 1. The deep learning model was constructed using the TensorFlow framework (1.15.0). Learning was performed using an NVIDIA Titan RTX graphics card of 24 GB. The batch size was set to six, and the learning procedure was completed in approximately 100 epochs.

### Evaluation

Segmentation was evaluated using dice similarity coefficient (DSC) values. DSC comparatively analyzed the ground truth and the predicted values. It was obtained by determining the ratio of the overlapped area between the predicted value and the ground truth with the sum of the predicted value and the ground truth. A value closer to 1 indicated the accuracy of the method. DSC was calculated as follows:

$$DSC (V_{seg} , V_{gs} ) = \frac{{2\left| {V_{seg} \cap V_{gs} } \right|}}{{\left| {V_{seg} } \right| + \left| {V_{gs} } \right|}},$$

where V_gs_ was the volume for the ground truth, and V_seg_ was the segmented volume.

In addition, the time required for segmentation of each dataset was measured at each training step. The time required for the training step was automatically measured and recorded on AVIEW Modeler software (version 1.0.3, Coreline Software, Seoul, Korea). Furthermore, a comparative evaluation of the accuracy between the manual and active learning segmentation obtained for the internal and external data was performed. For visual scoring, because intuitive scoring of IAN is difficult with 2D images, 3D scoring was performed using the AVIEW modeler software. The visual scoring evaluation was performed considering a grading scale, where four points were assigned for very accurate cases and one point for inaccurate cases. For visual scoring, three dentists conducted grade examination (manual ground truth *vs.* deep learning). The mean grade was calculated by the three dentists as follows. Grade 4 (Very accurate): when the labelled IAN completely matches the original IAN (over 95%); Grade 3 (Accurate): when the labelled IAN almost completely matches the original IAN (85%–9585–95%); Grade 2 (Mostly accurate): when the labelled IAN depicts the site of the original IAN (over 50%); Grade 1 (Inaccurate): when the labelled IAN depicts outside of the IAN or only matches a small area of the original IAN (under 50%).

## Results

**Fig. 3:**
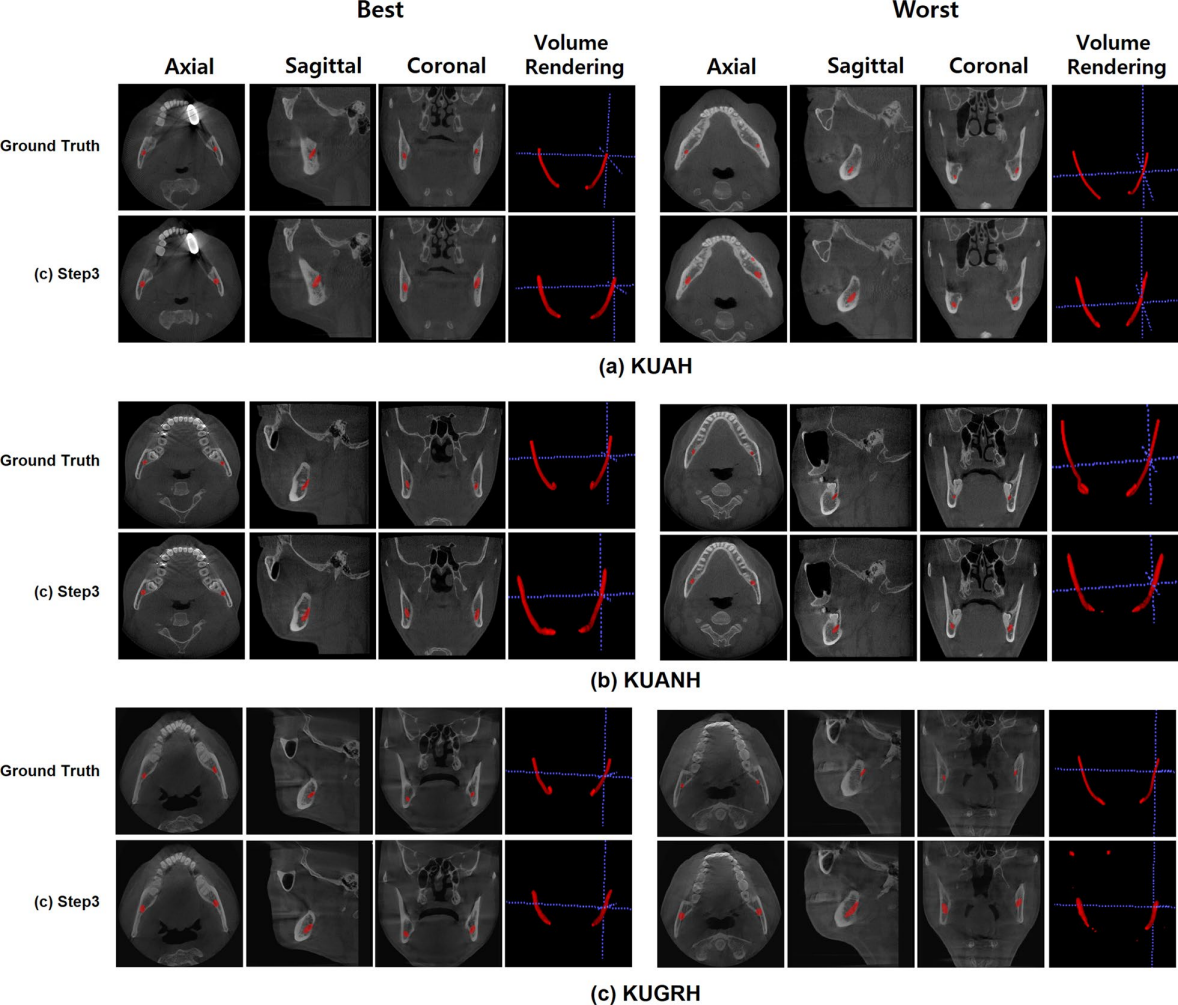
High-performance and low-performance segmentation results from the internal and external test dataset

In this study, we validated the effect of active learning using a customized nnU-Net. The segmentation results improved as the steps progressed. The average DSC value for the IAN segmentation gradually increased after each learning step, with values of 0.48 ± 0.11, 0.50 ± 0.11, and 0.58 ± 0.08 (Mean ± SD), respectively, and showed the best results in the last step (Table 2). When accuracy was evaluated using an external dataset after learning, the DSC value for data obtained from B was 0.55 ± 0.11, and that for the data from C was 0.43 ± 0.13 (Table 3). The low-performing and high-performing IAN segmentation results for data obtained from A, B, and C were depicted (Fig. 3).

**Table 2:** Dice similarity coefficients after automatic segmentation of inferior alveolar nerve at the first, second, and last steps for the internal dataset (83 cases)

Mean ± SD (range)	First step	Second step	Last step
DSC	0.48 ± 0.11 (0.26–0.62)	0.50 ± 0.11 (0.32–0.62)	0.58 ± 0.08 (0.39–0.65)

DSC, dice similarity coefficient; SD, standard deviation

**Table 3:** Dice similarity coefficients after automatic segmentation of inferior alveolar nerve in the test dataset [Internal: 15 cases (A), External: 20 cases each (B and C)]

Mean ± SD (range)	Last step (A)	Last step (B)	Last step (C)
DSC	0.58 ± 0.08 (0.39–0.65)	0.55 ± 0.10 (0.39–0.69)	0.43 ± 0.13 (0.0–0.65)

DSC, dice similarity coefficient; A, Korea University Anam Hospital; B, Korea University Ansan Hospital; C, Korea University Guro Hospital; SD, standard deviation

The average time required for segmentation of each dataset was 124.8 s for manual partitioning in the first step and 143.4 s for manual correction after CNN-assistance in the second step. In the last step, after CNN-assistance, the time required for segmentation improved to 86.4 s after manual modification (Table 4). Further, in comparison to the first manual segment and the second stage, the time required decreased by approximately 38.4 s and 57.0 s, respectively.

**Table 4:** Comparison of segmentation time for each dataset between the manual and convolutional neural network-assisted and manually modified segmentation approaches

	First step	Second step	Last step
	Manual segmentation	CNN-assisted and manually modified segmentation	CNN-assisted and manually modified segmentation
Average time (s)	124.8 ± 54.18	143.4 ± 102.66	86.4 ± 61.8

CNN, convolutional neural network

The visual scoring results were presented in Table 5. It was observed that the accuracy of manual segmentation was higher than that of automatic segmentation.

**Table 5:** Qualitative results from visual scoring of automatic inferior alveolar nerve segmentation on cone beam computed tomography from 53 randomly selected data (Internal: A, External: B and C)

Grade	Manual						nnU-net (Last step of active learning)					
	A		B		C		A		B		C	
	Rt	Lt	Rt	Lt	Rt	Lt	Rt	Lt	Rt	Lt	Rt	Lt
4 (very accurate)	14.3	14.3	19	19	19	19	11	9.7	10.7	12.3	5.3	4.7
3 (accurate)	0.7	0.7	0	0	0	0	2.7	3	7.3	5	8.7	7
2 (mostly accurate)	0	0	0	0	0	0	1.3	2	1	1.4	3.3	6.3
1 (inaccurate)	0	0	0	0	0	0	0	0.3	0	0.3	1.7	1

A, Korea University Anam Hospital; B, Korea University Ansan Hospital; C, Korea University Guro Hospital; Rt, right; Lt, left

## Discussion

Determining the exact location of the IAN is an important step in planning and realizing the treatment of the oral and maxillofacial areas using tools such as extraction, dental implants, and orthognathic surgery [1]. In general, the position of the IAN is designated and labeled manually in each section of the CBCT image. However, there are some limitations in manual IAN labeling using CBCT. First, it is difficult to accurately measure density and because of noise in the images [15]. Second, since the IAN travels in various directions in 3D, it is difficult to intuitively determine the exact shape [16]. Thirdly, it is difficult to determine whether the shape or position of the nerve has been deformed due to adjacent teeth or lesions [17]. Finally, if the cortical layer of the mandibular canal surrounding the IAN is unclear from the image, it is difficult to determine the exact location [18]. Due to these limitations, manually labelling an image slice can be time-consuming.

Therefore, in this study, a step-wise customized nnU-Net method that enabled active learning with limited data was implemented. AI-based convenient labeling of IANs increases training efficiency. In fact, the overall improvement in the performance was observed after gradual refinements of AI with deep learning. As each step was completed, the DSC increased, and the segmentation time gradually decreased; approximately two-thirds of segmentation time were accomplished comparable to the manual segmentation.

In addition, in comparison to other studies, a multi-center dataset was used. The fact that using datasets from various institutions was a unique feature of this study that was different from other studies. The spectral structure of the trained data and the spectral structure of actual clinical data may be different, so external validation was mandatory in AI research [19]. It was demonstrated that AI performance can be improved through diversification of the dataset. Since the multi-center dataset was not utilized during learning, diversity in the AI learning stage could not be implemented. However, by comparing the segmentation efficiency of datasets acquired using equipment from different companies and specifications at different hospitals, it was possible to identify areas that need improvement through further study. Additionally, the consistency of the study model was confirmed with visual scoring of the results from each institution.

In this study, a customized nnU-Net method was used as the network. The nnU-Net method is derived from a 3D U-net and batch normalization [13]. 3D U-Net is a widely used method for image segmentation, but its overall performance may be affected by CNN structure, pre-processing, and learning [20]. In contrast, nnU-Net is based on 3D U-Net, but it is more robust and shows more stable and improved performance owing to the self-adapting framework [13]. In the SegNet method, the decoder upsamples the functional map using the maximum pooling index instead of learning similar to that in a fully convolutional network (FCN) [21]. In contrast, the U-net up sampling operation learns to deconvolute the input function map, outputs it to the decoder, and combines it with the high-resolution function map [22]. In addition, as an extension of the 2D layer, a 3D layer was trained by randomly selecting 64 3D patches with 320 × 320 pixel images. The 3D network has the advantage of maintaining valid padding with the original 3D architecture, as it obtains more contextual information [23].

Previous studies on IAN segmentation with deep learning have provided considerable insights. Kwak et al. achieved an accuracy of about 0.82–0.99 in IAN segmentation using 102 CBCT datasets, 2D SegNet, and 2D and 3D U-Nets [24]. Jaskari et al. observed DSC values of about 0.57 to 0.58 using 637 CBCT datasets and 3D U-Nets [25]. Vinayahalingam et al. used 82 panoramic images and 2D U-Net and observed a DSC value of approximately 0.77–0.80 [26]. Overall, our model performance was similar to that of previous AI models in literature.

In an author’s previous study about automatic segmentation of the lesion and air of the maxillary sinus, which was conducted in the same way as this study, the DSC of the lesion of the maxillary sinus obtained through learning was ~ 0.760, and that of the air was ~ 0.930, while the segmentation time was ~ 362 s. Thus, in comparison to the manual segmentation a higher DSC and faster segmentation were achieved [14]. For IAN segmentation conducted in this study, a DSC of ~ 0.58 and a segmentation time of ~ 86.4 s were observed, which were not as high as the maxillary sinus segmentation data. Presumably, in the maxillary sinus image, the difference in the Hounsfield unit (HU) inside and outside the segmentation area was prominent, resulting in a lower error value during segmentation. However, considering the IAN, the difference in HU between the inner and outer bone marrow in the mandibular canal is relatively small, which can result in errors during segmentation. Indeed, according to the literature, the HU in the mandibular canal is -726.4, and the HU in the cancellous bone is 416.2 [27]. Additionally, during IAN segmentation, a shorter time was observed. However, we need to consider a larger number of image cuts for comparison.

**Fig. 4:**
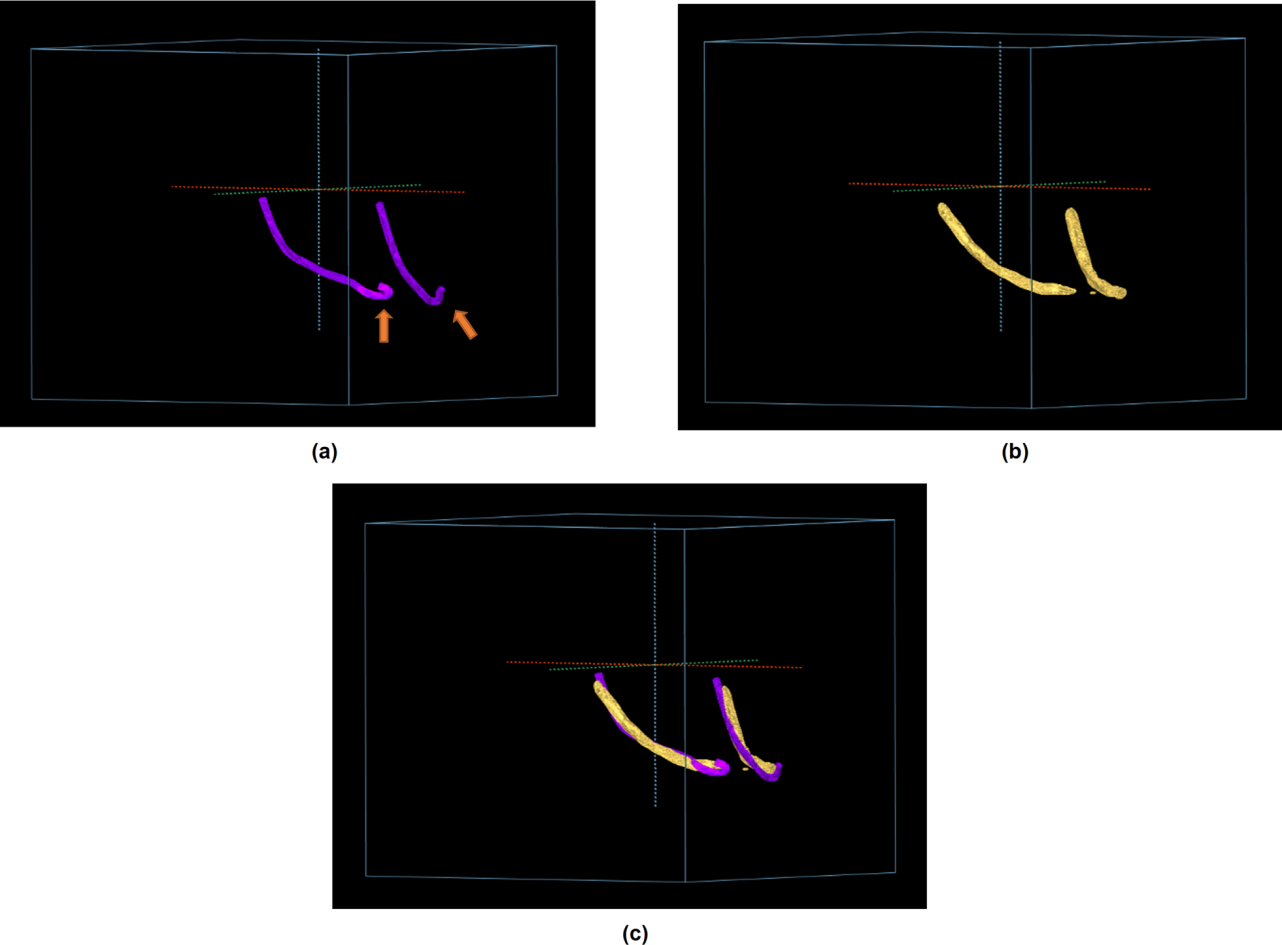
Improperly implemented anterior loop shape of the inferior alveolar nerve during deep learning

Another limitation of this study is that the shape of the anterior loop, which is partly inside the mental foramen at the end of the IAN, was not well implemented in deep learning (Fig. 4). We presume that due to the incomplete manual segmentation input data for learning, our model accuracy was lower. Thus, improvements in this direction should improve the accuracy of automatic IAN segmentation.

For the model developed in this study, we believe that effective learning is necessary to overcome the difference in segmentation efficiency for each anatomical application site. In addition, the initial input value i.e., the manual segmentation dataset, should be more precise to improve the accuracy. Moreover, the amount and diversity of the training dataset should be increased, and the segmentation performance should be improved using more diverse networks during training. Furthermore, additional multi-center datasets and comparisons with other segmented networks, such as cascade networks, must be performed to further verify the efficiency and stability of this methodology.

Manual IAN labeling is unintuitive, exhausting, and inconsistent, and it may not always be precise or consistent. Labeling using active learning in future will surely replace existing methods. Therefore, the active learning framework presented in this study is a robust tool that can support manual labeling by improving the accuracy and reducing the procedural time.

## Conclusion

Through this study, we confirmed that the deep active learning framework can improve the labeling accuracy of the IAN. Further, efficient learning on the limited CBCT dataset reduces the segmentation time, which can be implemented as a convenient automatic IAN labeling technique for future clinical use.

## Data Availability

The datasets generated and/or analysed during the current study are not publicly available due [REASON WHY DATA ARE NOT PUBLIC] but are available from the corresponding author on reasonable request.

## Abbreviations

2D: Two-dimensional
3D: Three-dimensional
AI: Artificial intelligence
CBCT: Cone-beam computed tomography
CNN: Convolutional neural network
DSC: Dice similarity coefficient
HU: Hounsfield unit
IAN: Inferior alveolar nerve
A: Korea University Anam Hospital
B: Korea University Ansan Hospital
C: Korea University Guro Hospital
ReLU: Random rectified linear unit
SSM: Statistical shape model
